# Student perspectives of simulated learning to improve their dysphagia management

**DOI:** 10.4102/sajcd.v71i1.1060

**Published:** 2024-09-30

**Authors:** Skye N. Adams, Kelly-Ann Kater, Jaishika Seedat

**Affiliations:** 1 Department of Speech Pathology and Audiology, School of Human and Community Development, University of the Witwatersrand, Johannesburg, South Africa

**Keywords:** simulation learning, clinical skills, soft skills, self-reflection, undergraduate education

## Abstract

**Background:**

The use of simulation to enhance knowledge translation and bridge the theoretical-clinical gap to enhance clinical training and competency in health professions has received mixed reviews in the literature.

**Objectives:**

This research examined student perspectives of a simulation laboratory in speech therapy to improve students’ clinical competency when working with adults with communication and dysphagia impairments.

**Method:**

An exploratory descriptive pilot study was conducted in 2022 with 16 third-year speech-language therapy students. This mixed-methods study involved students completing purposefully developed pre-and post-surveys to explore their experiences with simulated teaching and learning and their perceptions of confidence. Data were analysed using an independent *t*-test. Following the surveys, the students participated in a focus group discussion about their simulation experience, and data were analysed using thematic analysis.

**Results:**

Student ratings of clinical skills improved from pre to post-simulation significantly overall and across six out of the eight items. The focus group revealed insights into students’ experiences, highlighting increased confidence, the benefits of making mistakes in a safe environment and improved preparedness to work with dysphagia in patients.

**Conclusion:**

While simulation serves as a valuable tool in enhancing clinical skills and building confidence, it must be used as an adjunct to real-life exposure and not as a replacement.

**Contribution:**

The integration of both simulated and real-life experiences is essential to provide a comprehensive and practical learning environment for students.

## Introduction

Simulation is gaining recognition as an effective approach for developing and evaluating practical skills in the area of dysphagia and within the medical field (Broadfoot & Estis, 2020; Buléon et al., 2023; Hewat et al., 2020). Speech-language therapists (SLTs) play crucial roles in the management of dysphagia, making it a vital area of study for students in both fields (Howells et al., 2019; Saleem et al., 2022). Speech-language therapists focus on the rehabilitative aspect of dysphagia management through the assessment and treatment of swallowing disorders by devising and implementing therapy strategies to improve swallowing mechanics and safety (ASHA, 2002). The absence of standardised training programmes to manage dysphagia has resulted in varied levels of competency and preparedness (Coutts, 2019; Singh et al., 2015).

Research has found that traditional teaching methods have been important for the foundational theoretical skills but insufficient in preparing students to handle complex clinical cases (Smith & Doe, 2020). Rising student enrolments and practical constraints have amplified the demand for alternative clinical training modalities (Coutts & Barber, 2023; Seedat et al., 2021). There have been a growing number of students and graduates in rehabilitation sciences in South Africa (Ned et al., 2020; Pillay et al., 2020) that has resulted in increased pressures on institutions to provide more hands-on dysphagia training than before. Simulated learning environments (SLEs) have become a practical solution for enhancing clinical and soft skills alongside traditional teaching methods and have shown promising results in bridging the gap between theoretical knowledge and practical application (Mass-Ramírez et al., 2021) with majority of research coming from countries such as New Zealand, Australia and the United States (US). Hill et al. (2021) suggested that SLEs may replace traditional clinical training methods and still achieve the same competencies. This research shows that SLEs not only helped students develop essential clinical skills but also improved their confidence in handling complex cases. In addition, Rose et al. (2017) reported statistically significant increases in student self-reported levels of confidence, knowledge and experience when working in acute care.

While the benefits of SLEs have been explored in medical and nursing education, their application in speech-language therapy remains under-explored, particularly across different geographical contexts and student populations. In regions such as South Africa, factors such as cultural differences, resource availability and varying educational standards may significantly influence the effectiveness of SLEs (Bowen-Withington et al., 2020; Mills et al., 2020; Penman et al., 2020). Cultural factors can affect the relevance and authenticity of simulated scenarios, while resource limitations, such as restricted access to high-quality simulation technology, may create disparities in learning experiences. Moreover, the cost of maintaining and updating simulation equipment, alongside the need for specialised instructor training, can pose further challenges to the widespread adoption of SLEs (AlGerafi et al., 2023; Chernikova et al., 2020; Ward et al., 2023). Studying SLEs in the South African context and other low-resource settings is crucial to understanding how these barriers can be addressed and how SLEs can be utilised or adapted to improve training in diverse, real-world environments.

The Speech-Language Therapy Department at the University of the Witwatersrand is the first speech-language therapy department in Africa, tailored for developing a wide range of clinical skills. The simulation lab was to serve as a space for skill development, simulating a spectrum of clinical scenarios. It was viewed as a safe environment where students could engage in deliberate practice, receive immediate feedback and iteratively refine their techniques without the consequences of real-life clinical interventions. Recognising speech-language therapy as an inherently practical profession, the department acknowledges the common challenges students face in translating theoretical knowledge from lectures into effective clinical practice (Hill et al., 2021) Challenges in securing workplace clinical training and access to patients, along with difficulties in providing safe learning environments that allow for task repetition by students, have made simulated learning a viable option for inclusion in undergraduate university curricula (Hewat et al., 2020; Snowdon et al., 2020). Simulated learning is underpinned by practice-based pedagogies that prioritise experiential learning and reflective practice (Owen, 2016; Raelin, 2008).

The need for improved preparation of students and new graduates to deliver effective dysphagia care is well-documented, both globally and within South Africa (Caesar & Kitila, 2020; Singh et al., 2015). Despite achieving minimum competencies, many new clinicians feel ill-prepared for the complexities of dysphagia, necessitating enhanced training approaches that address these deficiencies (Coutts, 2019; Singh et al., 2015). Given the limited training opportunities in public healthcare settings (Caesar & Kitila, 2020), SLEs present a feasible alternative for meeting the comprehensive needs of students, educators and patients. Therefore, the simulation lab serves three primary purposes: facilitating the transition from academic study to clinical application, enhancing clinical proficiency in a dynamic setting and cultivating essential soft skills needed for patient interactions.

This study aimed to explore speech-language therapy students’ perceptions of a simulation-based learning experience using an adult mannequin with dysphagia because of neurological impairment. The research focussed on assessing students’ non-technical skills, particularly their confidence and preparedness in conducting dysphagia assessments, both before and after the simulation. The findings aim to refine simulated patient scenarios, increase student engagement and enhance skill acquisition and self-confidence in managing neurogenic communication and swallowing disorders.

## Research methods and design

### Study design and participants

This study employed an explanatory sequential mixed-methods design. In the first phase, 16 third-year speech-language therapy students participated in a training programme and completed pre- and post-surveys to assess their experiences, confidence and preparedness in clinical skills for dysphagia assessment. The second phase involved qualitative data collection through in-person focus group discussions (FGDs), with students divided into two groups of eight. These discussions provided detailed insights into their simulation experiences. The combined quantitative and qualitative data aimed to refine simulated patient scenarios, enhance student engagement and improve skill acquisition and self-esteem in managing dysphagia. The criteria for student inclusion were as follows: students had to be enrolled in the speech-language therapy degree at the University of the Witwatersrand, be in their third-year of study and be assigned to a clinical placement working with either adults or children with dysphagia. Third-year students were chosen as participants because they had recently completed the adult dysphagia theory course.

#### Recruitment

There was a 61.5% participation of all the eligible third-year speech-language therapy students (*n* = 16). Emails were sent to the third-year cohort (*n* = 26) and the study was discussed in face-to-face sessions. All students provided written informed consent. The study took place between September 2022 and December 2022. Participation was voluntary and the researchers confirmed to students that there will be no penalties for non-participation or benefits for participation. Typical traditional clinical placements would continue. All students were also provided with a code that was used throughout the research process to ensure confidentiality and anonymous processing of the data.

### Procedure

#### Development of the scenarios

**FIGURE 1:**
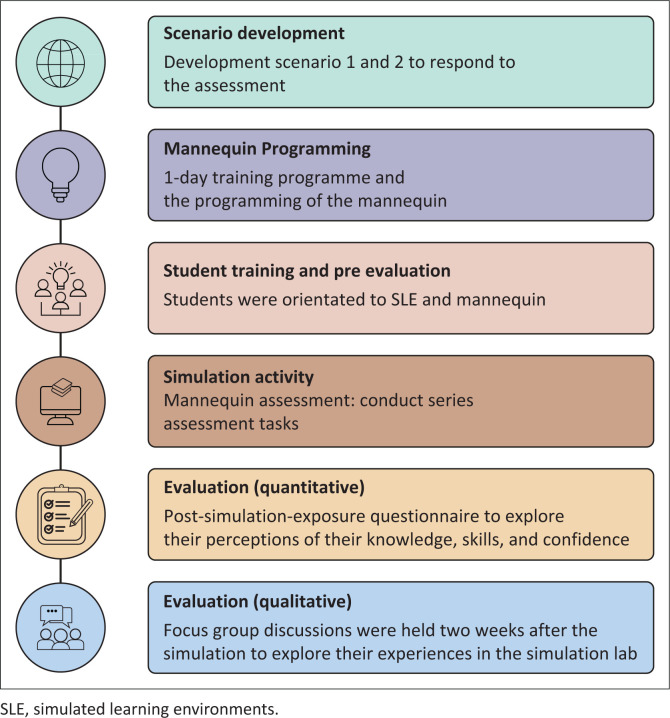
Research process of the simulation training and activity.

The researchers developed scenarios and then used these to programme the mannequin (Figure 1). The scenarios chosen were typical of the cases in hospitals in South Africa. These included traumatic brain injury (TBI) (resulting from motor vehicle accidents) and stroke, both of which have high prevalence rates in South Africa (Ackah et al., 2021). Dysphagia was the primary focus of the scenarios; however, speech and language aspects could not be ignored as both stroke and TBI typically include speech, language and swallowing difficulties. This encouraged the student to look at the patient (i.e., the mannequin) holistically.

**Scenario 1**: A 50-year-old patient, following a middle cerebral artery (MCA) stroke, presented at the emergency department of a hospital with right-sided weakness and slurred speech. He was admitted into the neurology ward. He was seen by a neurologist, physiotherapist and occupational therapist. He was referred to the SLT by the physiotherapist as he was presenting with a weak cough and crepitus bilaterally.

**Scenario 2:** A 37-year-old patient who was involved in a violent, high-speed motor vehicle accident, was in the trauma ward for 4 days before being transferred to a high-care medical ward. He had a computed tomography (CT) of the brain which showed a lesion on the brainstem located on the right side of the medulla. The researchers programmed the mannequin’s vital signs and asked the students to observe these as they entered the room. The mannequin was programmed to respond to questions asked by the student such as to orientation to person, place and time as well as a receptive and expressive language screener. Overt signs of dysphagia, such as coughing, choking, throat clearing and changes in vital signs were also programmed into the mannequin. In cases where the mannequin could not respond, the researcher provided the student with an explanation of how the mannequin would typically respond at that moment. For example, the mannequin presented with anterior spillage after the swallow, this would be described to the student to then proceed with the appropriate clinical response. Additionally, students were also required to role-play with the researchers to explain their diagnosis to the patient or provide feedback on their management to another healthcare provider.

#### Mannequin programming

The researchers who are all speech-language therapy academics with clinical and teaching experience in adult communication and swallowing disorders designed a framework for the simulation. Prior to the simulation activity, the researchers facilitating the simulation activities completed a 1-day training programme focussed on general principles of teaching using simulation and specific information about the simulations being used. The advanced HAL^®^ S3201 Multipurpose Patient Simulator from Gaumard Scientific was used in the current study. The researchers programmed the mannequin’s vital signs and pre-recorded the mannequin’s response to different questions that could be asked by the students such as orientation and answers to the speech and language screener (Supplementary material 1). Unfortunately, the mannequin did not display any physical characteristics such as facial weakness or drooling and these were all described to the student by the facilitator.

### Simulation activities

Prior to training within the SLE, student participants received training sessions on the orientation to the SLE, expectations, learning outcomes and an orientation to the simulation. Specific learning objectives linked to student learning level and existing knowledge and skills were developed and mapped to learning outcomes linked to SLT competencies. Table 1 outlines the areas of simulation that were targeted in the SLE workshop. Students were all under the supervision of a researcher, all of whom were SLTs working in the field of dysphagia. This model was based on previous research (Ward et al., 2015) as a way to guide students through the assessment and to assist students with their clinical decision-making skills in dysphagia management.

**TABLE 1:** Areas of simulation used in simulated learning environments in the field of dysphagia.

Simulations used	Areas of practice	Skills targeted
Mannequins Simulated environment Standardised patients	Adult communication Adult dysphagia	Case file review skills Communication skills Professional conduct skills Assessment skills Clinical reasoning skills Treatment planning skills

*Source*: Adapted from Macbean, N., Theodoros, D., Davidson, B., & Hill, A.E. (2013). Simulated learning environments in speech-language pathology: An Australian response. *International Journal of Speech-Language Pathology, 15*(3), 345–357. https://doi.org/10.3109/17549507.2013.779024

The students had two SLE training sessions with each focussing on one of the two scenarios. Students came into the simulation lab one at a time and commenced assessing the ‘patient’ – the student assumed the role of the SLT as they would in a typical clinical placement. At each session, there were two researchers present. Researcher one was responsible for the scenario presentation to the student and the second researcher controlled the mannequin and provided responses from the programmed mannequin.

Upon entering the simulation lab for the first time, students were orientated to the room, the mannequin and the vital signs monitors by researcher one. Following the orientation, students were required to conduct a series of assessment tasks based on the provided clinical data and medical history of the patient, including: (1) practise core clinical skills on speech, language and dysphagia assessments, (2) trial communication exchanges with patients (case history, information provision, confirmation of information), (3) conduct a file review (and decipher significant versus extraneous information), (4) conduct SLT dysphagia practice including assessment (oral motor sensory examination, receptive language, expressive language and a bedside dysphagia assessment), and (5) intervention and communication with the multidisciplinary team members (role played by a researcher). Students were informed that all tasks were timed to align with the fast-paced reality of the hospital context but to still provide a safe learning environment. Each student was given 15 min for their first scenario and 10 min for their second scenario. The similarity of the SLE to the typical hospital ward ended here in that unlike the scenario within a typical hospital ward, the SLE experience was undergirded by a dialogic process between the student (SLT) and the researcher (clinical educator). During this process, the student received feedback and was asked to justify their choices and decisions. It was critical that the simulated environment provided a space for learning, questioning, feedback and discussion of the student’s clinical choices as part of the assessment.

For the dysphagia assessment, students were unable to give the mannequin any food or liquid and were again described what they would do. Students were required to assess all consistencies and the mannequin was programmed to provide different responses. When the mannequin had liquids they coughed consistently and showed changes in the oxygen saturation and respiratory rates, indicative of aspiration. The mannequin was programmed to present with silent aspiration when given semi-solids with oxygen saturation dropping and a respiratory rate increase which the students were asked to monitor when they gave the patient liquids and semi-solids. At the end of the assessment, the student was required to write notes in the patient file and provide feedback to the patient and nurse and/or doctor (actor). Students were also provided with immediate feedback on their responses and performance by the researchers.

### Evaluation procedures

Students were required to complete a pre-and post-simulation-exposure questionnaire (see Table 2) to explore their perceptions of their attitude and perceived skills and confidence levels following their experience in the simulation lab. Questionnaires were developed prior to the research. Although there was no formal validation process for the pre-and post-simulation questionnaires, they were designed based on established literature and expert input to ensure they addressed key areas of student confidence, preparedness and perceived skill acquisition related to dysphagia management. All responses provided were confidential; however, to ensure surveys could be linked to surveys pre-and post-exposure, they all had to enter their unique identifier on each survey. Students were asked a number of questions before and after the simulation lab workshop to determine the change in their attitude using a pre-and post-Likert scale from 1 to 5.

**TABLE 2:** Differences between pre-and post-test of attitudes and perceived confidence towards assessment of dysphagia using a simulated learning environment (*N* = 16).

Items	Pre-test	Post-test	Differences	*t*	*p*
	M ± s.d.	M ± s.d.	M ± s.d.		
1. I feel nervous to assess and/or manage a patient with dysphagia	4.25 ± 0.68	2.4 ± 0.96	1.81 ± 0.28	7.39	< 0.01
2. I feel confident to assess and/or manage a patient with dysphagia	1.94 ± 0.68	3.6 ± 0.9	1.63 ± 0.22	−9.04	< 0.01
3. I am excited to assess and manage patients with dysphagia	4 ± 0.73	3.44 ± 0.96	0.56 ± 0.23	2.05	0.06
4. I am not looking forward to assessing or managing a patient with dysphagia	1.88 ± 0.96	1.5 ± 0.51	0.37 ± 0.44	1.69	0.11
5. I believe I have the clinical skills I need to assess and manage a patient with dysphagia	2.4 ± 0.73	4.06 ± 0.77	1.63 ± 0.04	−7.34	< 0.01
6. I feel competent to assess and manage a patient with dysphagia	2.5 ± 0.97	4.12 ± 0.81	1.63 ± 0.16	−5.96	< 0.01
7. I feel anxious about going to the hospital to assess and/or manage a patient with dysphagia for the first time in fourth year	4.4 ± 0.72	2.29 ± 1.05	2.19 ± 0.33	7.61	< 0.01
8. I am well-prepared to work in the hospital when I get to fourth year, when I start seeing patients with dysphagia.	2.2 ± 0.83	3.79 ± 0.7	1.69 ± 0.12	−7.83	< 0.01

**Total average**	**2.9 ± 0.78**	**3.12 ± 0.74**	**1.43 ± 0.22**	**-2.46**	**0.026**

Note rows highlighted in grey = statistically significant (*p* < 0.01).

M, mean; s.d., standard deviation.

Two weeks after the simulation lab workshop, all students attended a FGD to discuss their experiences in the simulation lab as well as any recommendations they would make. Students were placed into two focus groups (*n* = 8) to discuss the use of the simulation lab. Lecturers in the degree were excluded from the evaluation aspects of the study to eliminate bias but were involved in the training in the simulation lab.

### Data analysis

The quantitative data was processed and summarised using Statistical Package for the Social Sciences (SPSS) 23.0, which included the calculation and reporting of mean values and standard deviations for the responses. All ratings were made using 5-point equal appearing interval scales, encompassing semantic descriptors that ranged from strong agreement to strong disagreement, with ‘not sure’ serving as the neutral midpoint. Students were required to provide additional context for their ratings allowing for qualitative feedback. Data analysis included the calculation and reporting of mean values and standard deviations for the responses. To explore potential statistically significant variations in responses between the two groups of students, an independent *t*-test was performed, and the significance level was established at *p* < 0.05.

The qualitative data collected from the focus groups were analysed using inductive thematic analysis. All transcripts were imported into the computer program NVivo for coding and analysing the text content. A framework method was used to systematically categorise and code the interview transcripts and ensure rigour and quality of the analytical process (Ritchie et al., 2003; Saldaña, 2009). Qualitative findings were presented with baseline and post-simulation lab exposure findings.

### Ethical considerations

Ethical approval was obtained from the University of the Witwatersrand Human Research Ethics Committee (Non-medical) (Approval number: H22/03/12).

## Results

### Evaluation of the learning experience: Quantitative results

Students were asked to complete a pre-and post-simulation lab exposure questionnaire to determine their attitudes of dysphagia and their perceived confidence in assessing and managing dysphagia. The *t*-test results show a statistically significant difference between the overall pre-test and post-test scores (*t* = -2.46, *p* = 0.026). This suggests that the simulation activity had a significant impact on the students’ scores. Additionally, six out of the eight items showed significant differences. The item with the highest score after the simulation exposure was ‘I feel competent to assess and manage a patient with dysphagia’ (4.12 ± 0.81), while the lowest score was ‘I am not looking forward to assessing or managing a patient with dysphagia’ (1.5 ± 0.51) (Table 2).

### Evaluation of the learning experience and response attitudes: Qualitative results

Three main themes emerged from the content analysis of the FGDs. The themes are presented from most to least prevalent: (1) increased confidence, (2) simulation lab as an adjunct to real-life exposure, and (3) recommendations for a SLE (Table 3).

**TABLE 3:** Themes that emerged from the focus group discussions.

Theme	Sub-theme
1. Increased confidence	1.1 Making mistakes
2. Simulation lab as an adjunct to real-life exposure	2.1 Improved perceived competency in dysphagia
3. Recommendations for a simulated learning environment	3.1 Simulation to mimic real-life 3.2 Inclusion of multidisciplinary team members

#### Increased confidence

All students spoke about the increased confidence that the simulation lab provided as described by Participant 5, ‘It helped us to understand that we can do this, it is not as scary as it seems’ and Participant 9, ‘It helped with our confidence and then that also helped us with our clinical skills and you are kind of afforded the opportunity to make a mistake’. Students felt an increase in confidence as it reduced their anxieties and provided them with opportunities to practise and make mistakes. Making mistakes is an important process in student learning as described by Participant 11, ‘Because we could make those mistakes and learn from them we built on our knowledge and it mainly helped us to build our confidence’, and Participant 15:

‘Because having the skills is okay, but having the confidence for me to execute those skills is what is really important. I feel like confidence is a key factor in what all of us need to execute what we have learnt.’ (P15)

Students felt the exposure to the simulation lab, in particular for dysphagia, was important as they felt that it differed in terms of complexity and being able to be prepared more than other aspects of SLT training such as language intervention.

**Making mistakes and receiving feedback:** Students reported an increase in confidence because of the increased exposure to dysphagia and the ability to make mistakes, which are not often afforded in traditional clinic placements, as described by Participant 7:

‘I think what I enjoyed about it is that the environment that was created. So, you would like to explain why you’re doing something, and if you weren’t doing something correctly, or if you could have done something better. You kind of got feedback back straight away. And you’re kind of we’re afforded the opportunity almost to make a mistake.’ (P7)

Students appreciated the constant and immediate feedback. Immediate feedback is an important aspect in adult learning and training of professionals in a clinical setting (Irons & Elkington, 2021). Students felt that the clinical educator was much more relaxed in the SLE which allowed them to not feel as though they were unable to ask questions or try again as reported by Participant 4:

‘At the clinic my clinical educator kind of put me down and criticised me because I was unsure about the assessment and that made me feel very negative about dysphagia and scared that I would do the wrong thing and hurt somebody but the sim(ulation) lab gave me an opportunity to try again and ask questions.’ (P4)

A similar experience was reported by Participant 5 regarding the immediate feedback and positive influence on the student’s confidence:

‘The clinical educator provided immediate feedback and I felt like I was allowed to ask and say I don’t know because it wasn’t a real person sitting there. It just does build on your confidence, and it does make you realise that you do know what you’re doing and it’s just a great place to learn and to grow, and I think having confidence is very key in this profession. So, I think it really grew my confidence. And yeah, it just gave us an opportunity to learn and to learn from our mistakes and to receive feedback.’ (P5)

#### Simulation lab as an adjunct to real-life exposure

Many students brought up the limitations of the simulation lab such as the inability to see the patients’ physical characteristics or the environment that does not always mimic real-life regarding the busyness and interruptions or even patient responses that are not always as structured as they were in the simulation. Students reported on the difficulty in assessing dysphagia without being able to observe specific dysphagic symptoms such as facial weakness, pocketing or drooling as explained by Participant 8, ‘There was right-sided weakness but we didn’t actually see that’, and Participant 11, ‘So, we couldn’t know for example like what pocketing looks like, we couldn’t see that and didn’t get that exposure’.

Students highlighted that the simulation lab provided a safe learning environment, allowing them to focus on dysphagia without the distractions of a hospital setting. As Participant 8 stated:

‘[*T*]he hospital environment is stressful, and I think the sim lab was beneficial in the way that it gave you an opportunity to focus on dysphagia assessment, especially as new clinicians with no experience.’ (P8)

However, students also noted limitations of the simulation environment, specifically its inability to replicate the noises and distractions present in a real hospital, as mentioned by Participant 9:

‘[*I*]n the hospital setting its, really noisy and there’s people walking in and out and there is a lot of distraction, and yeah people shouting and the sim lab environment didn’t replicate that.’ (P9)

Participant 11 furthermore stated:

‘I’ve been in the hospital and it gets very noisy, and then you also have the physio come in and try and work with the client opposite me, and it does get distracting.’ (P11)

As a result, it is important that the simulation lab is used as an adjunct to real-life exposure and not in isolation. It is important to acknowledge the limitations that the simulation lab poses and the ways in which it is unable to simulate all real-life clinical noises, distractions and interactions.

**Improved perceived competency in dysphagia:** Students reported on their perceived improved competencies and feeling more equipped with specific dysphagia skills such as reviewing a patient file, understanding the application of international dysphagia diet standardisation initiative (IDDSI), monitoring oxygen saturation and aspiration and feeling a better sense of being able to integrate their theoretical and clinical skills. Participant 11 spoke about her improved understanding of how to go through a medical file:

‘We actually learnt how to look at like what to look for in the file, and like where to look, how to understand certain things, and then like moving on to the next steps.’ (P11)

Participant 14 also spoke about how she perceived an improvement in her overall dysphagia competencies because of the simulation lab exposure:

‘… it looked like a hospital room the files were there the stats were there. It felt like as real as it could get, and therefore, like I felt like I like I was doing … like I’m an actual speech-language pathologist like I knew what I was doing. So, before that I just had the theory, and I was like, oh, we have to do this we have to do this, IDDSI one IDDSI zero it’s like that sort of thing I didn’t know I just knew I had to do it. Now I know why I must do it depending on the patient, that’s sort of thing.’ (P14)

It is important to note that while students may have felt more equipped and competent, their actual competencies were not assessed. Furthermore, findings did not assess long-term retention of skills or transfer to real clinical settings.

#### Recommendations for a simulated learning environment

Students spoke about different ways the simulation lab experience could be improved to enhance their learning, including the imitation of real-life and working in a hospital setting, as well as the importance of working in a multidisciplinary team to improve learning outcomes.

**Simulation to mimic real-life:** Students commented on the importance of not focussing on dysphagia in isolation but to include other aspects such as linguistic, cognitive or cultural factors that would mimic real-life. Students brought up the fact that when working with real patients, some would speak different languages, ‘Our patient almost sounded Afrikaans but there weren’t any other languages used which I think would be a reality in our real-life’ (Participant 13) or would present with different conditions and not only dysphagia:

‘[*P*]atients generally don’t have one condition at a time. So we were focusing on a patient with a stroke, but so many patients with a TBI have a hearing problem and may have to use AAC, I felt that was where the sim lab fell short because it focused on one condition.’ (P4)

This was reiterated by Participant 7:

‘If we could see a patient more holistically it’s not just coming in about their dysphagia, but also to think about language and their discourse, their orientation, and their speech and all of that.’ (P7)

**Inclusion of multidisciplinary team members:** Students commented on the importance of viewing a patient holistically and the inclusion of other team members in the simulations, ‘I think having an opportunity to kind of see how it would work in an MDT. I think that would be quite cool’ (Participant 2). Participant 4 reiterated this and spoke about the importance of working within a multidisciplinary team and being able to communicate with other members of the team, such as nurses:

‘Like working in an MDT so like when we have to refer. I remember once, I recommended an NGT, and the nurse didn’t want to, she didn’t agree with the recommendation of putting the patient on a NGT. So, I think more of that could also be quite beneficial because sometimes we think it’s quite easy, and we just say refer or recommend something, but sometimes it’s not as simple in the real-world.’ (P4)

Working in a team is an important aspect of care, and the inclusion of other healthcare professionals could improve learning as well as exposure to other members of the rehabilitation team. Simulations using mannequins effectively foster multidisciplinary teamwork by providing a controlled, safe environment for practice, especially with healthcare professionals they may not have worked with before (Saragih et al., 2024). Additionally, simulations can be tailored to specific learning objectives and offer a realism that surpasses traditional role play, although there are still limitations when using mannequins for dysphagia (Chernikova et al., 2020).

## Discussion

Following participation in the simulated learning experience, students reported having a better understanding of working with patients with dysphagia and communication impairments. They also noted increased knowledge, experience and confidence in assessing patients with these conditions, which they felt could be effectively transferred to real-world settings. Students found the SLE exposure facilitated translation of their theoretical knowledge into practical skill implementation not just in dysphagia but more broadly in a conducive, uplifting and capacity building way that was not possible when ‘learning’ in a real-life situation. In the current study, students noted that they had the opportunity to do things again or say ‘I don’t know’ as there was not a real patient present. These findings are in line with previous research and the positive impact of utilising a SLE for dysphagia (Hill et al., 2014; Rose et al., 2017; Van Vuuren, 2016). The implications for the incorporation of SLEs in the education and training for dysphagia in South African programmes would be a positive step forward for transformative training of speech-language therapy graduates within the current geographical space given the many challenges faced with clinical training. However, it is important to address the pitfalls and limitations with this kind of training as mentioned by the students and the ways in which we can incorporate aspects of simulation that are more accessible in our context. In the current study, the experience of the students in the simulation lab was a positive one. While students acknowledged that lectures were instrumental in acquiring theoretical knowledge, a minority noted significant improvements in their skills or confidence through this mode of learning alone. However, after the simulation lab exposure, students reported that they felt they had the appropriate attitudes, skills and confidence to work with dysphagia patients in a hospital setting. The findings of this study align with those of Ward et al. (2015), highlighting the critical importance of integrating both lectures and simulated learning into student training to enhance clinical outcomes.

The findings showed that SLEs also provide students with an opportunity to repeatedly practise procedures, manage different clinical scenarios and make mistakes without real-world consequences, which is an invaluable part of the learning process. Studies conducted by Macbean et al. (2013) and Mills et al. (2020) also report that SLEs can enhance learning experiences by addressing the clinical placement challenges and evolving governing body requirements faced by many universities globally. These challenges include meeting the required number of clinical hours and providing students with exposure to a variety of patients with different diagnoses. In addition, research has also indicated that SLEs facilitate both development and refinement of clinical skills while reducing students’ anxiety and improve on their knowledge and communication skills before working with real patients (Quail et al., 2016; Rose et al., 2017; Van Vuuren, 2016). Therefore, the adoption of SLEs in medical and rehabilitative education in South Africa is not only a response to logistical challenges but also an innovative step towards a more equitable and comprehensive education model that prepares students for the diverse needs of the communities they will serve.

The response attitudes of speech-language therapy students measured before and after the simulation workshop showed an increase in the overall mean value. However, some items did show a statistically significant difference, which is in line with results from previous research (Kim & Lee, 2020; Sumbane et al., 2023; Van Vuuren, 2016). The nature of the questions asked pre-and post-SLE also may have contributed to this differing finding. Ward et al. (2015) asked specific dysphagia practice-related questions, whereas the questions in the current study were broad. Those questions that did not offer significant changes were around excitedness and eagerness to begin managing patients with dysphagia, which is understandable. These questions may have influenced the extent and scope of reporting in student confidence pre-and post-SLE. Students’ reported increases in confidence were corroborated with the qualitative findings and students’ increase in confidence when working with patients with dysphagia following simulation lab exposure confirmed the effectiveness of the experience and the need for future research to determine students’ learning of specific knowledge using the mannequin. Furthermore, it is important to note the study’s reliance on self-reported measures of confidence and competence, as students may overestimate their abilities following the workshop. For future studies, the inclusion of skills-based assessment should be considered to improve methodological rigour (Miles et al., 2016).

The learning experience in this study provided students with valuable advantages through additional time and feedback, and resources not consistently available during traditional clinical placements. Although this benefit is not directly related to the mannequin SLE, but rather to the enhanced supervision provided in this session, it underscores the importance of time and feedback in clinical education. Ideally, these elements should be integral to all clinical placements. While one-on-one training in the SLE is not realistic or feasible for all settings, providing high-quality feedback is imperative for effective adult learning (Irons & Elkington, 2021). Existing research underscores the significance of offering feedback to enhance student learning and development, a principle especially crucial for students from diverse backgrounds (Adamson et al., 2018; Attrill et al., 2012; Mupawose et al., 2021). Furthermore, delivering immediate verbal feedback has been proven to enhance clinical skills and bolster student confidence (Ho & Whitehill, 2009). Verbal feedback fosters open discussion, clarifications and in-depth exploration – essential elements of reflective practice. This bears weight in preparing students for clinical work and calls for a re-evaluation of our teaching methods and the types of support and feedback being provided to students.

Students emphasised the significance of involving other team members and striving for a more authentic representation of the clinical environment. Research has indicated that simulation is a way to facilitate and support multidisciplinary teamwork and a way to better mimic the clinical setting and the development of soft skills (Hamada et al., 2020; Smeets et al., 2023; Taylor et al., 2018; Zackoff et al., 2014). Unfortunately, these objectives present challenges within the constraints of a simulation lab, as it cannot fully replicate the unstructured and unpredictable interactions that typically occur in real-life patient encounters (Bressmann & Eriks-Brophy, 2012). These challenges were faced with the current study as well. It is important to acknowledge the inherent limitations of simulation labs and the importance of supplementing this training with exposure to genuine patient interactions, as previously discussed in this context. One viable approach might be to introduce not only mannequins but to use real actors and role play, as suggested by Bressmann and Eriks-Brophy (2012). Real-life actors could offer more realistic interactions, especially when dealing with challenging patients and incorporating physical attributes that mannequins cannot simulate, such as drooling and facial weakness.

The current study also reiterated the importance of both technical and non-technical skills in the assessment of dysphagia. While the current study focussed on the non-technical skills that is confidence, anxiety and self-assurance of the clinician (student), the intertwining effect of one on the other was noted and inevitable. The results confirmed that the SLE provided the context, opportunity and space for students to gain confidence and feel less anxious when faced with a patient with dysphagia, and these feelings, in turn, enabled them to be more intentional in their assessment of dysphagia. These findings are similar to other SLEs done at other sites in Queensland University (Australia), Auckland (New Zealand) and University of Florida (US), where simulation improves non-technical skills as a result of the simulation (Hill et al., 2021; Mills et al., 2020; Rose et al., 2017; Taylor et al., 2018; Ward et al., 2015). Caution is, however, necessary to ensure that an outcome of an overconfident student is not the result; hence, the SLE is proposed as an adjunct and bridge in the learning, implementation and experience continuum of the student in dysphagia but can also be used to improve both technical and non-technical skills (Bartlett et al., 2021).

Opportunities for low-stakes repeated practice assisted students in feeling more prepared for their clinical hospital placement. This study also incorporated aspects of role play and one-on-one learning with a lecturer, accompanied by immediate feedback. It is important to note that SLEs can often be resource and time-intensive and that this is not feasible for many institutions, particularly in low-middle income countries. Previous studies have also commented on the cost associated with simulation and the utilisation of high-tech mannequins (Ward et al., 2023). Therefore, it is important to extrapolate key elements into clinical practicums which could then significantly enhance the training experience and improve clinical competency. An example of this is to provide simulations without mannequins and instead to use actors who can be coached to give specific real-life responses, that is facial expression or even drooling that a mannequin is unable to produce. Simulations could also be incorporated into a peer learning model where students in the higher years of study or clinical educators could facilitate sessions for those in the earlier years to reduce the academic load. As noted by Hill et al. (2021), simulation can be used to supplement a portion of clinical training and should be used as an adjunct to traditional clinical placements that allow for students to practise their dysphagia skills in a conducive environment that they can grow positively and engage without fear.

### Limitations and future research

There are several limitations to this study and these will also be used to highlight possible opportunities for future research. Firstly, this study did not utilise a control group which may limit our ability to direct comparisons and draw definitive conclusions about the impact of the simulation lab for student training in dysphagia. However, our user experience indicates that it was still beneficial to students’ learning despite not having a control. Secondly, the simulation lab has provided opportunities for lecturers, clinicians and students working in the field of dysphagia in a low-stakes environment. However, training and skills are required in the development of other scenarios to ensure optimal use of hi-tech mannequins. The mannequins have a number of limitations in the field of dysphagia and should be used in conjunction with actors where more practical application of skills can be utilised. Lastly, as we transition towards a more multidisciplinary approach in teaching, clinical practice, and research, it becomes increasingly essential to incorporate this perspective into undergraduate training.Research has indicated that SLEs can play a pivotal role in enhancing interprofessional training for students. They provide a valuable opportunity for students to gain insights into different professions and understand how various disciplines can collaborate effectively in the assessment and management of patients, and it is an important avenue to explore (Lewis et al., 2017).

Future research could include a follow-up evaluation in the clinical setting or work place in order to ascertain the generalisation of skills from the simulation lab into clinical practice. Clinical training in South Africa has numerous challenges, and the implementation of SLEs may eliminate some of these. Therefore, it would be beneficial to analyse the cost-effectiveness of implementing SLEs in educational programmes and healthcare settings. Future research should also explore long-term learning outcomes in order to investigate the long-term impact of SLEs on student knowledge retention, clinical competence and professional development in the areas of dysphagia and communication. Recommendations are to refine the simulation experience based on student feedback to enhance the practical utility of the findings in order to determine the skills and knowledge transfer in specific caseloads using the SLE. In addition, it would be important to also explore the supervisor experience using the SLE. Ongoing research is needed to ensure that simulated teaching and learning does improve areas of student competency in areas beyond dysphagia.

## Conclusion

The findings of this study suggest that incorporating simulation focussed on adult communication and swallowing disorders can replace a portion of traditional clinical placement time for students. Previous research has shown that simulation is highly valued by students and contributes to confidence-building as well as knowledge and skill development. The results of the current study provide support for the use of SLEs at the ways in which they can be integrated into student teaching and learning, by offering a high-quality learning experience for students and potentially addressing the current demands on traditional clinical placements.

## Figures and Tables

**SCENARIO 2:**
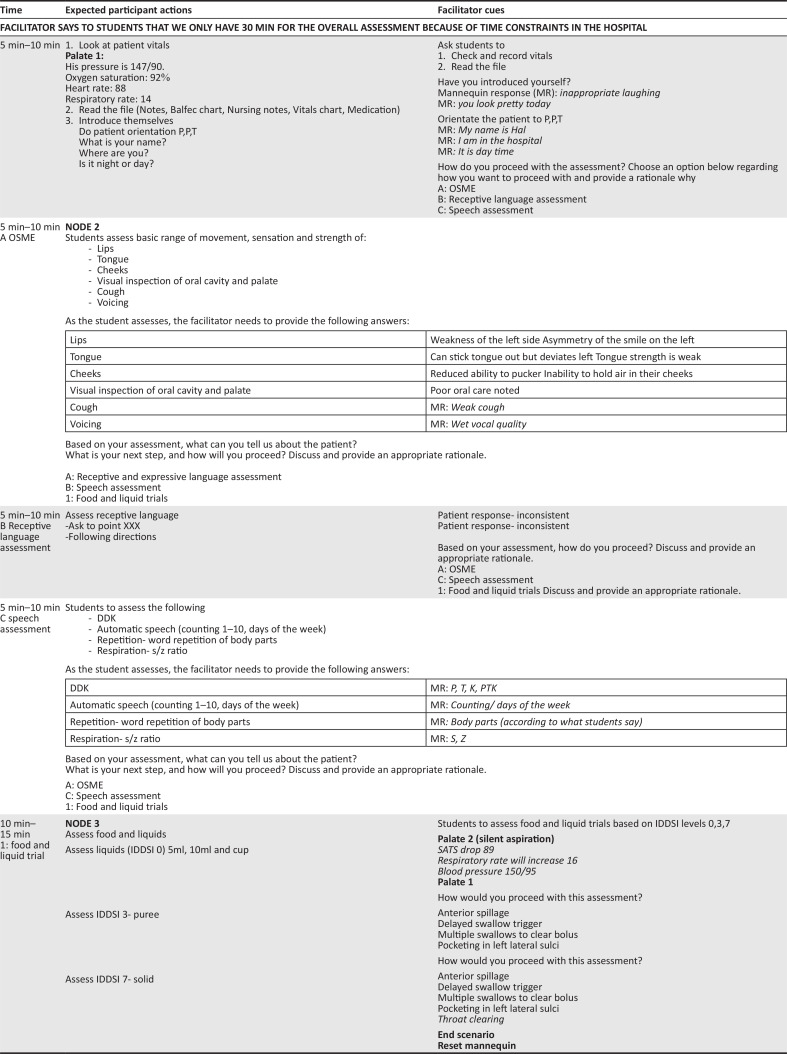
Simulated patient programme and script.
